# Prospective

**Published:** 1887-04

**Authors:** 


### PROSPECTIVE.

In the next number will be commenced a series of articles by Drs. Bödecker and Heitzmann, which will be continued through the year, we hope. The first articles of the series will be upon the development of Enamel, Dentine and Cementum. These will be followed by papers on Erosion and other equally interesting subjects, the titles of which it is not now necessary to announce. The first part is already in our hands, and judging from that, we believe that this will prove one of the most interesting and valuable series of articles yet presented to the dentists of America. The special qualifications of the authors for the task, no one will for a moment dispute. Whatever any one may think concerning the histological views of Dr. Heitzmann, he is admitted by all to be one of the most careful observers now engaged in special study, while in the ability to delineate with his pencil what the microscope reveals to him he stands unrivalled. Certainly, the engravings with which the series of articles will be profusely illustrated are the most exquisite of any that we ever beheld. They are all new and original, and are really valuable as works of art, aside from their technical importance.

Of the qualifications of Dr. Bödecker for writing a work of this kind, it is quite unnecessary for us to speak. The profession, not only of America, but of Europe as well, know him, and they know his untiring zeal and assiduity, and his high attainments in microscopical science, as well as in histological study. More, we need not say. Besides, we are quite sure to get a wigging for even these complimentary allusions, and if we were to say more it is possible that the modesty of Dr. Bödecker would not permit him to publish the articles at all.

We had expected to present the first part in this number, but some trifling defects in the engravings caught the critical eyes of the authors, and they were sent back for reproduction, and so reached our hands too late for use this month.

